# Evaluation of X-Ray Repair Cross-Complementing Family Members as Potential Biomarkers for Predicting Progression and Prognosis in Hepatocellular Carcinoma

**DOI:** 10.1155/2020/5751939

**Published:** 2020-03-17

**Authors:** Jie Mei, Huiyu Wang, Runjie Wang, Jiadong Pan, Chaoying Liu, Juanying Xu

**Affiliations:** Department of Oncology, Wuxi People's Hospital Affiliated to Nanjing Medical University, Wuxi 214023, China

## Abstract

The X-ray repair cross-complementing (XRCC) gene family has been revealed to participate in the carcinogenesis and development of numerous cancers. However, the expression profiles and prognostic values of XRCCs (XRCC1-6) in hepatocellular carcinoma (HCC) have not been explored up to now. The transcriptional levels of XRCCs in primary HCC tissues were analyzed by UALCAN and GEPIA. The relationship between XRCCs expression and HCC clinical characteristics was evaluated using UALCAN. Moreover, the prognostic values of XRCCs expression and mutations in HCC patients were investigated *via* the GEPIA and cBioPortal, respectively. Last but not least, the functions and pathways of XRCCs in HCC were also predicted by cBioPortal and DVAID. The transcriptional levels of all XRCCs in HCC tissues were notably elevated compared with normal liver tissues. Meanwhile, upregulated XRCCs expression was positively associated with clinical stages and tumor grades of HCC patients. Survival analysis using the GEPIA database revealed that high transcription levels of XRCC2/3/4/5/6 were associated with lower overall survival (OS) and high transcription levels of XRCC1/2/3/6 were correlated with poor disease-free survival (DFS) in HCC patients. Furthermore, Gene Ontology (GO) and Kyoto Encyclopedia of Genes and Genomes (KEGG) demonstrated the possible mechanisms of XRCCs and their associated genes participating in the oncogenesis of HCC. Our findings systematically elucidate the expression profiles and distinct prognostic values of XRCCs in HCC, which might provide promising therapeutic targets and novel prognostic biomarkers for HCC patients.

## 1. Introduction

Hepatocellular carcinoma (HCC) is one of the most fatal malignant tumors of the digestive system worldwide, which may cause 31,780 cancer-related deaths in the United States in 2019 according to the prediction by the American Cancer Society [1]. Although significant advances have been achieved in comprehensive treatment including surgery, chemotherapy, targeted therapy, and radiotherapy for HCC, the prognosis of patients with HCC remains largely unsatisfactory. Currently, the critical issue for HCC is the unfavorable five-year overall survival (OS) rate of only 30%–40% [2]. Besides, postoperative survival in patients with HCC varies widely. Detection of biomarkers associated with tumor malignancy and prognosis is critical for patients and clinicians. Although an increasing number of studies focusing on prognostic factors have been conducted [3, 4], it is still necessary to further explore more efficient potential biomarkers.

The X-ray repair cross-complementing (XRCC) gene family including numerous members (XRCC1-6, PRKDC, FANCG, BRCA2, etc.) mainly participates in homologous recombination to maintain chromosome stability and repair DNA damages. Several members have been reported to be involved in specific diseases. For example, FANCG plays a key role in the occurrence of Fanconi anemia [5]. Inherited mutations in BRCA1 and/or BRCA2 significantly increase the risks of breast cancer and ovarian cancer [6]. However, no literatures about the characteristic biological functions of six classical XRCCs genes (XRCC1, XRCC2, XRCC3, XRCC4, XRCC5, and XRCC6) are currently available. Dysregulation of XRCCs in cancerous disease may break repair processes and mechanisms of genetic instability, thus leading to tumorigenesis [7]. Consequently, our research focuses on the expression profiles and prognostic values of XRCCs in HCC.

With the successful implementation of numerous large-scale sequencing projects, including the Cancer Genome Atlas (TCGA) and the Genotype-Tissue Expression (GTEx), biomedical studies have been entering the field of “big data” [8–10]. Over the past few years, many interactive and user-friendly online platforms based on the TCGA database greatly elevate the efficiency of TCGA database analysis, and increasing amounts of tumor biomarkers have been identified based on the strength of these websites [11–13]. Our research employed these interactive online platforms to explore the expression profiles and prognostic values of XRCCs in HCC. Consequently, our research preliminarily and systematically summarizes the expression profiles of XRCCs in HCC and discusses the potential prognostic values of XRCCs expressions.

## 2. Materials and Methods

### 2.1. UALCAN

UALCAN (http://ualcan.path.uab.edu/) is an open-access platform based on level 3 RNA-seq and pathological files from the TCGA database [14]. It can be used to compare the relative transcriptional levels of candidate genes between tumor and paracancerous tissues as well as the correlation of genes mRNA levels with pathological features. In this research, UALCAN was employed to compare the transcriptional levels of XRCCs in primary HCC tissues and their association with pathological features.

### 2.2. GEPIA

Gene Expression Profiling Interactive Analysis (GEPIA, http://gepia.cancer-pku.cn/) is an interactive web server developed recently for analyzing the RNA-sequencing expression data as well as the association between gene expression and prognosis from the TCGA and the GTEx database [15]. The differential expressions of XRCCs in cancerous and adjacent tissues were validated, and the prognostic values of XRCCs at the mRNA level in HCC were analyzed by GEPIA. The patients' cohorts were split at the median expression of each XRCCs mRNA level. All cohorts were compared with Kaplan–Meier plots. Hazard ratio (HR) and log-rank *P* value were calculated and displayed online.

### 2.3. cBioPortal

cBioPortal (http://www.cbioportal.org/) is a user-friendly, interactive website and provides visualization, analysis, and download of large-scale cancer genomics datasets [16, 17]. In the current research, we analyzed the genetic alterations of XRCCs, which contained mutations and putative copy-number alterations from GISTIC. Furthermore, Genetic mutations in XRCCs and their association with OS and DFS of HCC patients were displayed online, and the log-rank test was performed to check the difference between different groups.

### 2.4. GO and KEGG Analysis

The Database for Annotation, Visualization and Integrated Discovery (DAVID, https://david.ncifcrf.gov/) [18] was employed to perform Gene Ontology (GO) and Kyoto Encyclopedia of Genes and Genomes (KEGG) analyses of six XRCC genes and the 36 most frequently altered neighboring genes. The human genome (Homo sapiens) was selected as the background variable.

### 2.5. Statistical Analysis

All statistical analyses were performed on the bioinformatics database online. The differential mRNA expression of XRCCs in HCC tissues was analyzed by Student's *t*-test. Kaplan–Meier survival plots were generated online with survival curves compared by log-rank test. For all analyses, differences were considered statistically significant if *P* values were less than 0.05.

## 3. Results

### 3.1. Upregulation of XRCCs in Patients with HCC

**Figure 1:**
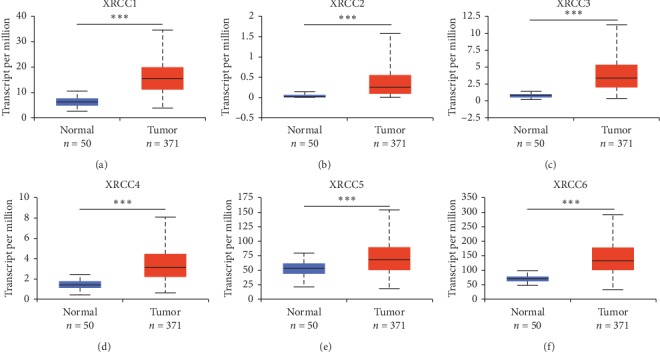
Transcriptional levels of XRCCs in paracancerous and HCC tissues (UALCAN). Comparison of XRCC1, XRCC2, XRCC3, XRCC4, XRCC5, and XRCC6 mRNA expression in paracancerous (*n* = 50) and HCC (*n* = 371) tissues in TCGA database based on data mining *via* UALCAN. The transcriptional levels of (a) XRCC1, (b) XRCC2, (c) XRCC3, (d) XRCC4, (e) XRCC5, and (f) XRCC6 were significantly upregulated in HCC tissues compared with paracancerous tissues. ^*∗∗∗*^*P* < 0.001.

**Figure 2:**
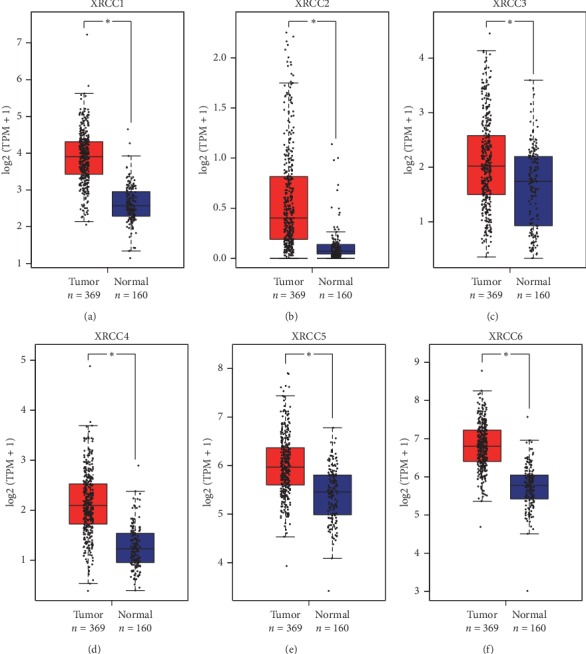
Transcriptional levels of XRCCs in paracancerous and HCC tissues (GEPIA). Validation of differential XRCCs expressions in paracancerous (*n* = 160) and HCC (*n* = 369) tissues in TCGA and GTEx dataset based on GEPIA. The transcriptional levels of (a) XRCC1, (b) XRCC2, (c) XRCC3, (d) XRCC4, (e) XRCC5, and (f) XRCC6 were remarkably upregulated in HCC tissues compared with paracancerous tissues. *P* cutoff: 0.001.

In order to assess the precise expression profiles of XRCCs in HCC samples, the differential transcriptional levels of XRCCs between HCC and normal liver tissues were evaluated using the UALCAN database. As shown in Figure 1, the transcriptional levels of all XRCC members (Figures 1(a)–1(f)) were notably upregulated in HCC tissues compared with paracancerous tissues. However, in consideration of the limited number of normal liver specimens in the TCGA database, we further employed the GEPIA web server containing more RNA-sequencing data of normal tissues from the GTEx database to confirm the differential expression of XRCCs in HCC. As we expected, the high transcriptional levels of XRCCs in HCC tissues were confirmed using the GEPIA website (Figures 2(a)–2(f)). Taken together, our results provided strong evidence showing that XRCCs were overexpressed in patients with HCC.

### 3.2. Association of mRNA Expression of XRCCs with Clinical Characteristics of HCC Patients

**Figure 3:**
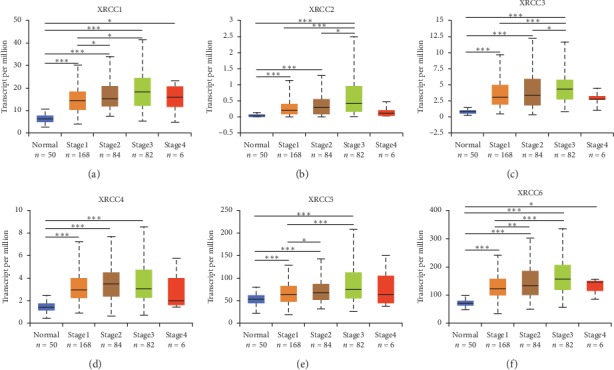
Relationship between mRNA expression of XRCCs and clinical stages of HCC patients. mRNA expressions of six XRCCs were remarkably correlated with patients' clinical stages; patients who were in advanced stages tended to express higher mRNA expression of XRCCs. (a–c, e, f) The highest mRNA expressions of XRCC1/2/3/5/6 were found in Stage 3, (d) while the highest mRNA expression of XRCC4 was found in Stage 2. ^*∗*^*P* < 0.05, ^*∗∗*^*P* < 0.01, ^*∗∗∗*^*P* < 0.001.

**Figure 4:**
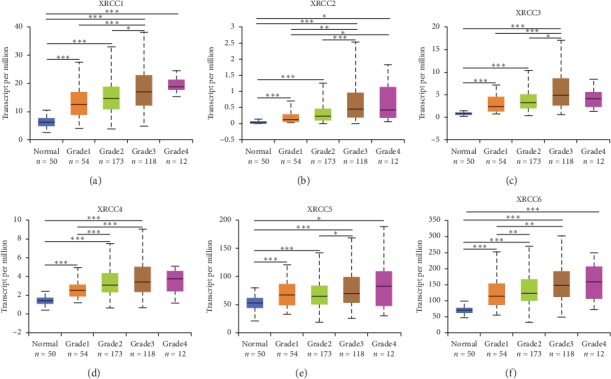
Association of mRNA expression of XRCCs with tumor grades of HCC patients. mRNA expressions of 6 XRCCs were significantly related to tumor grades, and as tumor grades increased, the mRNA expressions of XRCCs tended to be higher. (a, d–f) The highest mRNA expressions of XRCC1/4/5/6 were found in tumor Grade 4, (b, c) while the highest mRNA expression of XRCC2/3 was found in Grade 3. ^*∗*^*P* < 0.05, ^*∗∗*^*P* < 0.01, ^*∗∗∗*^*P* < 0.001.

After high expression of XRCCs was confirmed in HCC, we speculated that overexpression of XRCCs may correlate with the advanced clinical characteristics of HCC patients. So, we next analyzed the association between mRNA expression of XRCCs with clinical characteristics of HCC patients by UALCAN, including patients' clinical stages and tumor grades. As shown in Figure 3, mRNA expression of XRCCs was significantly correlated with advanced clinical stages, namely, patients who were with advanced clinical stages tended to express higher XRCCs mRNA. The highest mRNA expressions of XRCCs (excluding XRCC4) were found in Stage 3 (Figures 3(a)–3(f)), and the highest mRNA expressions of XRCC4 were found in Stage 2 (Figure 3(d)). The reason why the mRNA expressions of XRCCs in Stage 3 seemed to be higher than that in Stage 4 may be due to the limited number of Stage 4 patients (only 6 HCC patients were at Stage 4). Analogously, as shown in Figure 4, the mRNA expressions of six XRCCs were positively related to tumor grade. The highest mRNA expressions of XRCC1/4/5/6 were found in Grade 4 (Figures 4(a), 4(d)–4(f)), while the highest mRNA expression of XRCC2/3 was found in grade 3 (Figures 4(b) and 4(c)). Overall, these findings above implied that mRNA levels of XRCCs were significantly correlated with clinical characteristics in HCC patients and may serve as potential biomarkers for advanced HCC stages or poor differentiation.

### 3.3. Prognostic Values of XRCCs Expression in HCC Patients

**Figure 5:**
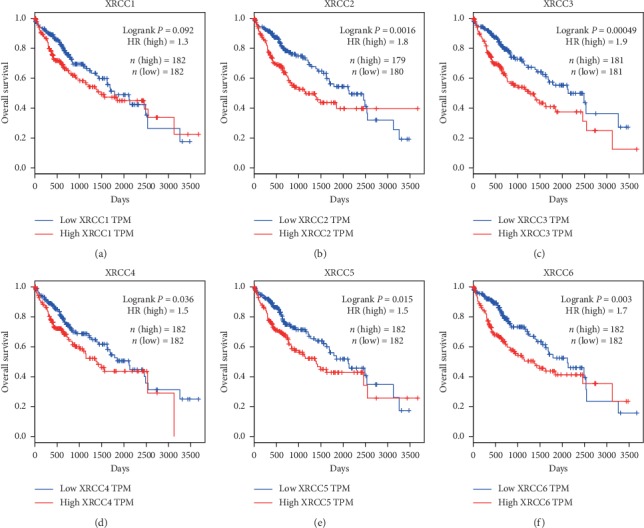
Prognostic value of XRCCs mRNA in HCC patients (OS). OS curves were plotted to evaluate the prognostic value of XRCCs mRNA expression. High mRNA expressions of (b) XRCC2, (c) XRCC3, (d) XRCC4, (e) XRCC5, and (f) XRCC6 were significantly associated with poor OS, while the expression of (a) XRCC1 had no association with OS of HCC patients.

Furthermore, we used the GEPIA website to evaluate the prognostic values of XRCCs. As shown in Figure 5, high mRNA expressions of XRCCs (excluding XRCC1) were all significantly associated with poor OS of HCC patients (Figures 5(b)–5(f)), while patients with high mRNA expression of XRCC1 also showed the trend with shorter OS (Figure 5(a)).

**Figure 6:**
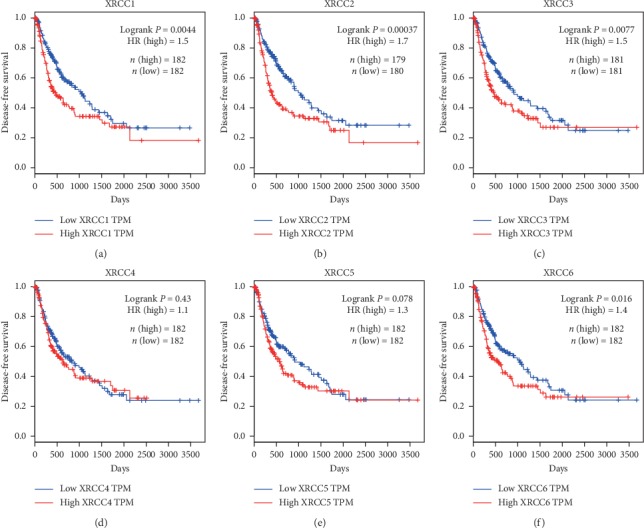
Prognostic value of XRCCs mRNA in HCC patients (RFS). RFS curves were plotted to evaluate the prognostic value of XRCCs mRNA expression. High mRNA expressions of (a) XRCC1, (b) XRCC2, (c) XRCC3, and (f) XRCC6 were remarkably associated with worse DFS, while the expression of (d) XRCC4, and (e) XRCC5 had no associations with DFS of HCC patients.

We next analyzed the associations between XRCCs mRNA expression and disease-free survival (DFS) of HCC patients, and the results exhibited that high mRNA expression of XRCC1/2/3/6 was significantly associated with shorter DFS of HCC patients (Figures 6(a)–6(c), 6(f)), while mRNA expression of XRCC4/5 showed no predictive values in estimating DFS of HCC patients (Figures 6(d) and 6(e)). To sum up, most XRCCs were associated with poor prognosis, which might be identified as promising biomarkers to predict the survival of HCC patients.

### 3.4. Genetic Alterations in XRCCs and Association with Prognosis of HCC Patients

**Figure 7:**
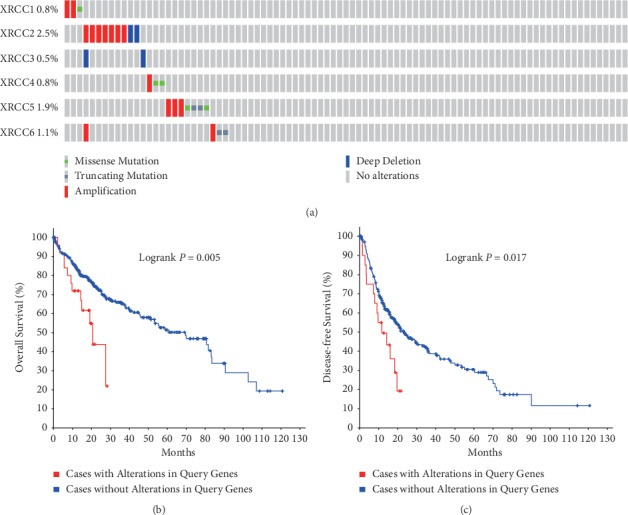
Correlation between the genetic alterations of XRCCs and prognosis of HCC patients. (a) OncoPrint in cBioPortal database exhibited the proportion and distribution of specimens with genetic alterations in XRCCs. Genetic alterations in XRCCs were notably associated with shorter (b) OS and (c) DFS of HCC patients.

Next, we analyzed genetic alterations in XRCCs and their associations with OS and DFS of HCC patients. As was shown in Figure 7, a low mutation rate of XRCCs was found in HCC patients. In the 366 sequenced HCC patients, the genetic alteration was found in only 27 HCC patients and the mutation rate was 7%. Although mutations in XRCCs were not frequent, genetic alterations were significantly associated with poor prognosis. Kaplan–Meier analysis showed that patients with genetic alterations in XRCCs had worse OS (Figure 7(b)) and DFS (Figure 7(c)) in HCC patients. These results revealed that genetic alterations of XRCCs could also notably affect HCC patients' prognosis.

### 3.5. Predicted Functions and Pathways of XRCCs and Their Frequently Altered Neighbor Genes in HCC Patients

**Figure 8:**
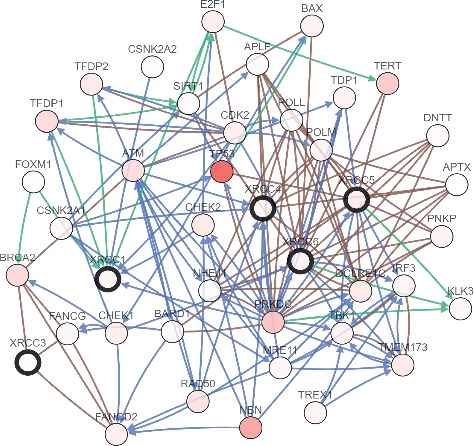
Predicted pathways of XRCCs and their 36 frequently altered neighbor genes in HCC patients. The network of XRCCs and their 36 frequently altered neighbor genes were constructed. The total 36 genes were frequently affected by XRCCs alterations.

**Figure 9:**
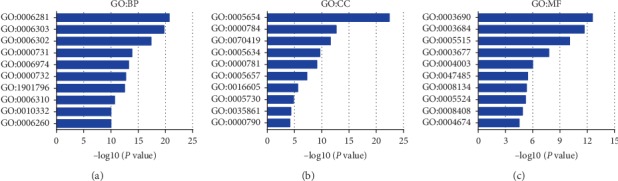
GO analysis of XRCCs and their associated genes. Gene Ontology (GO) enrichment analysis predicted the functional roles of target host genes based on three aspects including (a) biological processes (BP), (b) cellular components (CC), and (c) molecular functions (MF). Top 10 terms of BP, CC, and MF analysis were represented in this figure.

**Figure 10:**
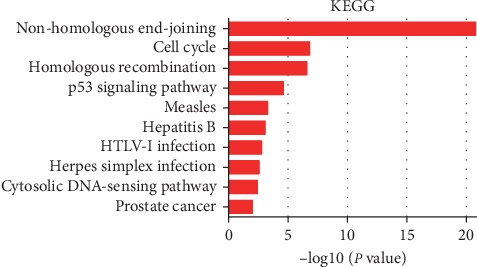
KEGG analysis of XRCCs and their associated genes. The functions of XRCCs and genes significantly associated with XRCCs alterations were predicted by analysis of the Kyoto Encyclopedia of Genes and Genomes (KEGG). The top 10 terms of KEGG analysis were represented in this figure.

All the results suggested that XRCCs may play the roles of significant oncogenes in HCC. Next, to explore the potential mechanisms that XRCCs participate in the carcinogenesis of HCC, we used cBioPortal to construct a network for XRCCs, and the results revealed that a total of 36 genes were significantly associated with XRCCs alterations (Figure 8). Moreover, GO and KEGG analyses based on DAVID were performed to identify the functional enrichment of XRCCs and their associated genes. GO analysis possessed three main functions of selected genes, including biological process (BP), cellular components (CC), and molecular functions (MF). We finally reserved the top 10 terms of every subanalysis, including BP, CC, MF, and KEGG (Figures 9(a)–9(c), 10). Overall, these findings suggested potential mechanisms of XRCCs participating in HCC oncogenesis, which established the foundation for the coming molecular mechanism research.

## 4. Discussion

Abnormality of cancer genetics is an intrinsic factor in tumorigenesis and has been found to participate in the development and progression of HCC [19]. External environmental factors play a role in the development of tumors by affecting the stability of related genes. Therefore, the stability of the gene, namely, the repair ability after DNA damage is closely related to the occurrence of the tumor, determines the difference in the susceptibility of different individuals to tumors. Being important components of DNA repair genes, XRCCs are involved in the development of numerous cancers, including HCC [20, 21]. Despite some members of XRCCs have been shown to play critical roles in HCC, the accurate roles of XRCCs in HCC remained to be explored.

In this research, the transcriptional expressions, genetic alterations, and prognostic values of XRCCs in HCC were analyzed. Our results exhibited that the upregulation of mRNA levels was found in all six XRCCs, and mRNA expression of XRCCs was remarkably correlated with patients' clinical stages and tumor grades in HCC patients. Besides, high transcription levels of XRCC2/3/4/5/6 were associated with lower OS and high transcription levels of XRCC1/2/3/6 were correlated with poor DFS in HCC patients. Moreover, mutations in XRCCs were not frequent, but genetic alterations were significantly associated with poor prognosis. Finally, Kaplan–Meier analysis exhibited that patients with genetic alterations of XRCCs genes had worse OS and DFS in HCC patients. All these findings suggested that XRCCs were essential for HCC oncogenesis and development.

As an effective analysis method, GO and KEGG analyses provide a comprehensive set of functional annotation approaches for investigators to understand the biological meaning behind the list of genes [22]. In our research, the functions and pathways of the alterations in XRCCs and their 36 frequently altered neighbor genes in HCC patients were analyzed, and our results suggested among all KEGG enriched terms, nonhomologous end-joining (NHEJ) was the most relevant terms. Chromosomal instability is a characteristic feature of HCC, Teoh et al. demonstrated that defects in the NHEJ DNA repair pathway may participate in the disruption of cell cycle checkpoints leading to chromosomal instability and accelerated development of HCC [23]. Besides, other enriched terms, including cell cycle, homologous recombination, and p53 signaling pathway , were also shown to affect HCC progression [24–26]. Taken together, these findings provide more in-depth insight on how XRCCs and these XRCC-related genes participate in HCC progression.

Among the XRCCs, XRCC1 is the most studied in cancerous disease. Overexpression of XRCC1 contributes to the development of ovarian cancer and its high expression was associated with advanced malignancy and poor clinical outcomes in ovarian cancer patients [27]. Besides, high expression of XRCC1 has also been found in glioma and gastric cancer [28, 29]. XRCC1 gene polymorphisms have been reported to be involved in multiple cancers, including breast cancer [30], lung cancer [31], and pancreatic cancer [32], but the potential mechanisms have not been identified. Meng et al. revealed a functional XRCC1 SNP, rs3213245, which enhances the risk of cervical cancer through mediating the Sp1/Krox-20 switch [33]. Moreover, XRCC1 interacts with ALDH2 and predicts poor OS in patients with lung cancer and liver cancer [34].

XRCC2 had been shown to participate in chemoresistance to 5-Fluorouracil in colorectal cancer [35]. An increasing number of studies observed the relationship between the polymorphisms in the XRCC2 gene and the risk of multiple cancers [36, 37]. However, recent research focused on the mechanisms that XRCC2 involved in the oncogenesis of cancer are not available yet. The association between the expression or polymorphisms of XRCC2 and the risk of HCC has no current reports as well.

Similar to XRCC1 and XRCC2, XRCC3 has been researched widely in the correlation between gene polymorphisms and the risk of cancer. Several studies have revealed multiple gene mutations, including rs861539 C > T, rs1799796 A > G, C241T, which are significantly associated with enhanced risk of HCC [38, 39]. Besides, XRCC3 overexpression has been found to be associated with clinical factors in breast cancer [40]. However, the expression profiles of XRCC3 in HCC have not been reported yet.

XRCC4, a member of XRCCs, had been reported to be an independent prognostic factor for HCC, and high XRCC4 expression was remarkably associated with HCC pathological features [21]. Genetic variants of XRCC4 were associated with susceptibility to esophageal cancer, and high expression of XRCC4 participated in radio-resistance in patients with esophageal cancer [41, 42]. Besides, XRCC4 depletion significantly sensitized cancer cells to chemotherapy and/or radiotherapy. Downregulation of XRCC4 by UHRF1 depletion sensitized retinoblastoma cells to more chemotherapy [43]. Silencing of XRCC4 increased the radio-sensitivity of breast cancer cells [44].

XRCC5 was found to promote the development of numerous cancers, including gastric cancer and colon cancer [45, 46]. Recent reports had also focused on the oncogene role of XRCC6. Zhu et al. revealed that high XRCC6 promoted the osteosarcoma process *via* the Wnt/*β*-catenin signaling pathway [47]. A literature review has been demonstrated the association between the XRCC5/XRCC6 dimer and the susceptibility to multiple cancers [48]. Besides, genetic polymorphisms in XRCC5 and XRCC6 genes also enhanced the risk of HCC [49].

Noticeably, there were some limitations to the current study. First, all the data involved in our study was obtained from online websites. Although the field of large data is the frontier of biomedical research, some unavoidable problems must be taken seriously. Mostly, sequencing databases, including TCGA and GTEx, only provide gene expression data at the mRNA level, which may not fully represent the expression even the activation of the candidate genes in the protein level. Further studies should apply western blotting as well as other protein detection techniques to validate our findings in protein level and explore the potential mechanisms of distinct XRCCs in HCC. Besides, further exploration of the clinical application of the XRCCs members in the targeted therapy of HCC should also be conducted in the future.

## 5. Conclusion

In summary, we systemically analyzed the expression profiles and prognostic values of XRCCs in HCC. Our results revealed that the overexpression of six XRCCs was found to be remarkably associated with clinical stages and tumor grades in HCC patients. Besides, higher mRNA expressions of XRCC2/3/4/5/6 were found to be significantly correlated with OS in HCC patients, while higher mRNA expressions of XRCC1/2/3/6 were notably correlated with favorable DFS. Overall, our research provided a systematic insight into the heterogeneous and complex roles of XRCCs in the carcinogenesis of HCC.

## Data Availability

The data used to support the findings of this study are available from the relative bioinformatics database.
